# *Momordica charantia* L.: Nutritional Composition, Advanced Extraction Methods, Phytochemistry, Molecular Mechanisms and Industrial Applications

**DOI:** 10.3390/antiox15070839

**Published:** 2026-07-02

**Authors:** Asad Abbas, Iqra Tabassum, Saeed Vohra, Ralf Weiskirchen, Areesha Shoukat, Muhammad Khurram Afzal, Adan Ijaz, Nimra Anees, Anis Ahmad Chaudhary, Abdulrahman Mohammed Alhudhaibi

**Affiliations:** 1 Multan College of Nutritional Sciences, Multan Medical and Dental College, Multan 60000, Pakistan; asadabbaskhichi@gmail.com (A.A.); iqra.it24@gmail.com (I.T.); areeshashoukat1@gmail.com (A.S.); adanijax@gmail.com (A.I.); 2 Department of Human Nutrition, Faculty of Food Science and Nutrition, Bahauddin Zakariya University, Multan 60800, Pakistan; khurram.afzal@bzu.edu.pk (M.K.A.); nimraanees2002@gmail.com (N.A.); 3 Department of Anatomy and Physiology, College of Medicine, Imam Mohammad Ibn Saud Islamic University, Riyadh 11623, Saudi Arabia; 4 Institute of Molecular Pathobiochemistry, Experimental Gene Therapy and Clinical Chemistry (IFMPEGKC), RWTH University Hospital Aachen, D-52074 Aachen, Germany; 5 Department of Biology, College of Science, Imam Mohammad Ibn Saud Islamic University (IMSIU), Riyadh 11623, Saudi Arabia; aachaudhary@imamu.edu.sa (A.A.C.); amalhudhaibi@imamu.edu.sa (A.M.A.)

**Keywords:** *Momordica charantia* L., nutritional composition, phytochemistry, advanced extraction techniques, molecular mechanism, industrial applications

## Abstract

*Momordica charantia* L. is a medicinal plant rich in bioactive compounds, including steroidal glycosides, flavonoids, phenolics, triterpenoids, saponins, and polysaccharides, which exhibit antidiabetic, antioxidant, anti-inflammatory, hepatoprotective, and anticancer activities. This review summarizes its nutritional and phytochemical composition, green extraction technologies, molecular mechanisms, and industrial applications based on literature from Google Scholar, PubMed, Scopus, Web of Science, ScienceDirect, and other scientific databases. Ultrasound-assisted extraction is an efficient and eco-friendly method that provides higher recovery of bioactive compounds from *M. charantia* and improved bioavailability compared with enzyme-assisted, microwave-assisted, and conventional methods. The phytochemicals of *M. charantia* regulate oxidative stress, inflammation, lipid peroxidation, and glucose homeostasis. Studies show that its antidiabetic effects involve improved insulin sensitivity, enhanced glucose uptake, and inhibition of carbohydrate-digesting enzymes. These compounds also exhibit antioxidant activity through free radical scavenging and anti-inflammatory effects via inhibition of the NF-κB and MAPK pathways. *M. charantia* further demonstrates anticancer activity by inducing apoptosis, causing cell-cycle arrest, and downregulating proliferation pathways in several cancer cell lines, including MCF-7, HCT-116, HepG2, A549, and PANC-1. Beyond medicinal uses, it is applied in the food industry as a functional ingredient in products such as yogurt, cookies, pickles, bread, juice, oil, and beverages. Overall, *M. charantia* shows strong potential for therapeutic applications, including functional foods and pharmaceutical formulations targeting diabetes, inflammation, liver diseases, and cancer; however, further studies are needed to confirm its clinical efficacy.

## 1. Introduction

In an era where chronic diseases such as cancer, diabetes, obesity, and cardiovascular disorders are rapidly increasing worldwide, the search for natural remedies that function as both food and medicine has become increasingly urgent. With the growing demand for sustainable health solutions, plants that provide both essential nutrition and therapeutic benefits have attracted significant attention for managing modern health challenges [1,2]. In this context, medicinal foods and edible plants with combined nutritional and pharmacological properties have emerged as promising candidates for functional foods, nutraceuticals, and pharmaceutical development [3]. *Momordica charantia* L. (*M. charantia*), commonly known as bitter gourd, bitter melon, balsam pear, karela, or bitter cucumber, occupies a prominent place in traditional medical systems across the globe, including Ayurveda and Unani medicine in the Indian subcontinent, Philippine folk medicine, traditional Chinese medicine, and the traditional pharmacopoeias of the Caribbean, Latin America, and sub-Saharan Africa [4,5].

*M. charantia* originated in the Indian subcontinent, where it subsequently spread across Asia, Africa, the Caribbean, and South America through ancient trade and colonial routes. Although initially cultivated as a food crop, its characteristic bitterness and bioactive cucurbitane-type triterpenoids facilitated its early adoption in traditional medicinal systems for the management of metabolic disorders, infectious diseases, and gastrointestinal conditions [6]. The immature fruit is the most commonly consumed part, while the leaves, vines, roots, seeds, and fruit juice are also used for the management of diabetes, fever, inflammation, jaundice, skin disorders, toothache, diarrhea, and infections. The plant is rich in minerals, vitamins, dietary fiber, and phytochemicals, including phenolics, flavonoids, alkaloids, bioactive peptides, polysaccharides, cucurbitane-type triterpenoids, steroidal saponins, and glycosides [7,8]. Charantin, a mixture of steroidal glucosides such as β-sitosterol glucoside and stigmasterol glucoside, is one of its best-known bioactive compounds due to its hypoglycemic, anticancer, anti-inflammatory, and hepatoprotective effects [9,10].

Recent scientific and industrial interest in *M. charantia* has focused on advanced extraction technologies, as conventional methods are often time-consuming, solvent-intensive, and less effective for thermolabile and complex phytochemicals [11,12]. Ultrasound-assisted extraction (UAE) is an emerging green extraction technique that reduces extraction time, enhances mass transfer, disrupts plant cell walls via acoustic cavitation, and improves the yield of phenolics, antioxidants, and charantin [13,14]. Despite these advantages, UAE presents several limitations, including challenges in scale-up due to non-uniform energy distribution, relatively high energy consumption at the industrial level, equipment costs, and variability in extraction efficiency depending on plant matrix composition, particle size, and solvent system [15]. Comparative studies have shown that microwave-assisted extraction often provides faster extraction kinetics and higher yields for intracellular compounds, although with a greater risk of thermal degradation if not carefully controlled [16].

In comparison, other advanced techniques remain less explored for *M. charantia*. Enzyme-assisted extraction (EAE) is still limited in application but offers mild conditions and high selectivity [17]. Supercritical fluid extraction (SFE), particularly using CO_2_, provides highly selective and clean extracts with minimal solvent residues, but its use is limited by high capital investment and operational complexity [18]. Similarly, pressurized liquid extraction (PLE) offers efficient and rapid recovery of bioactives under elevated temperature and pressure [19]. Among the above-mentioned advanced extraction methods, UAE therefore represents a promising approach for producing standardized bitter melon extracts for functional food, nutraceutical, and pharmaceutical applications [13]. These extracts contain bioactive compounds such as triterpenoids, saponins, flavonoids, alkaloids, polypeptides, and glycosidic steroids (e.g., charantin), which contribute to various pharmacological activities, including anticancer, anti-inflammatory, antidiabetic, and hepatoprotective effects [14,20,21,22].

Preclinical and clinical studies have demonstrated anticancer, anti-inflammatory, antidiabetic, and hepatoprotective properties of *M. charantia* bioactive compounds [14,21,22]. These compounds modulate key cell-signaling pathways involved in cancer progression and chronic inflammation, including nuclear factor kappa B (NF-κB), mitogen-activated protein kinase (MAPK), signal transducer and activator of transcription 3 (STAT3), cyclooxygenase-2 (COX-2), 5-lipoxygenase (5-LOX), and phosphatidylinositol 3-kinase/protein kinase B (PI3K/Akt) pathways [23,24]. Mechanistic studies further show that the anticancer peptide BG-4 isolated from *M. charantia* induces apoptosis in human colon cancer cells by reducing the expression of the anti-apoptotic protein Bcl-2, increasing pro-apoptotic Bax, activating caspase-3, and modulating cell-cycle regulators such as p21 and cyclin-dependent kinase 2 (CDK2) [24,25]. The anti-inflammatory activity of *M. charantia* is largely mediated through signaling pathways. For example, momordicine I suppresses pro-inflammatory cytokines and inhibits NF-κB signaling while also reducing tumor growth and inducing apoptosis in cancer cells [26]. Its antidiabetic effects involve suppression of MAPK and NF-κB signaling in pancreatic cells, stimulation of insulin secretion, improvement of insulin sensitivity, activation of the AMP-activated protein kinase (AMPK) pathway, and inhibition of key gluconeogenic enzymes such as fructose-1,6-bisphosphatase and glucose-6phosphatase [27].

Previous reviews on *M. charantia* have mainly focused on isolated aspects such as its nutritional composition, phytochemical compounds, or pharmacological activities, rather than providing a multidisciplinary perspective [4,6,9,28]. These studies have primarily emphasized either its disease-specific potential or its phytochemical characterization, with limited consideration of mechanistic pathways and in vivo and in vitro mechanisms, as well as industrial applications within a single framework. In contrast, the present review offers a more comprehensive and updated synthesis by simultaneously integrating nutritional value, phytochemistry, biological activities, and industrial applications including food and pharmaceutical applications. In recent years, increasing interest in *M. charantia* as a functional food ingredient has further expanded research into bioavailability enhancement, processing strategies, and delivery systems aimed at improving the stability, palatability, and shelf life of its bioactive compounds. Accordingly, this review systematically evaluates studies published between 2007 and 2026, with emphasis on the relationship between phytochemical composition and biological activities including antioxidant, anti-inflammatory, glucose-homeostasis, anticancer, and hepatoprotective activities mediated through signaling pathways such as MAPK, NF-κB, PI3K/Akt, and AMPK. Overall, this review provides a more holistic and updated framework for understanding the multifunctional potential of *M. charantia,* while also identifying critical research gaps to support its evidence-based use in functional foods and the development of novel pharmaceutical products.

## 2. Search Methodology

The literature was searched in peer-reviewed journals on *M. charantia* regarding its nutritional composition, advanced extraction techniques, phytochemistry, molecular mechanisms, and applications in the food and pharmaceutical industries. Data were collected from PubMed/MEDLINE, Scopus, ScienceDirect, Web of Science, Embase, Google Scholar, and the Cochrane Library. Peer-reviewed original research articles including preclinical, in vivo, in vitro studies, systematic reviews, meta-analyses, and book chapters published between January 2007 and April 2026 were included. The search strategy used MeSH terms and Boolean operators with keywords such as “*Momordica charantia*,” OR “bitter melon,” OR “bitter gourd,” OR “karela,” OR “nutritional composition,” OR “phytochemicals,” OR “bioactive compounds,” OR “extraction methods,” OR “ultrasound-assisted extraction, “OR “supercritical fluid extraction,” OR “anticancer,” OR “antidiabetic,” OR “anti-inflammatory,” OR “hepatoprotective,” OR “antioxidant,” OR “NF-κB,” OR “AMPK,”OR “molecular mechanisms,” OR “functional food,” OR “nutraceutical,” and “pharmacological activity.” This structured approach was adapted across databases with appropriate syntax modifications to maintain sensitivity and reproducibility.

**Figure 1:**
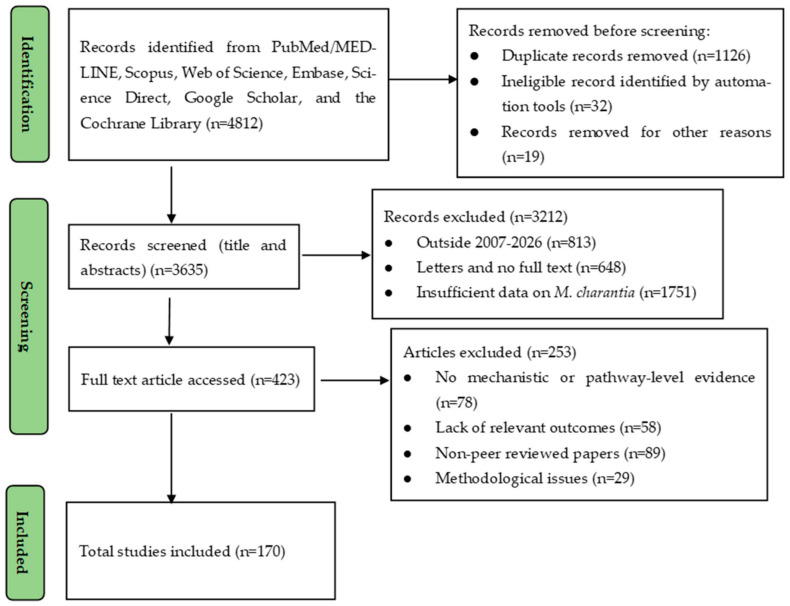
Literature search and study selection process.

All retrieved records were imported into a reference management system and screened in two stages based on predefined inclusion and exclusion criteria. First, titles and abstracts were screened to remove duplicates, records without full-text access, letters to the editor, and studies unrelated to *M. charantia* or outside the selected time frame (2007–2026). Second, the full texts of potentially relevant studies were evaluated for eligibility. Included studies reported (i) nutritional and/or phytochemical profiles of *M. charantia* parts (fruit, seeds, leaves, or pulp), (ii) extraction, isolation, or characterization of bioactive compounds, (iii) in vitro, in vivo, or clinical pharmacological effects with mechanistic or pathway-based evidence, or (iv) food industry or nutraceutical applications. In total, 170 peer-reviewed publications were included, covering nutritional characterization, phytochemical profiling, advanced extraction methods, biological mechanisms, and industrial applications of *M. charantia* as shown in Figure 1.

## 3. Nutritional Composition and Antioxidant Potential of Different Parts

Different parts of *M. charantia*, including leaves, seeds, fruits, and pulp, are rich in proteins, fats, carbohydrates, and essential minerals. The nutritional and phytochemical composition of these parts is summarized in Table 1. The seeds exhibit the highest nutritional value, containing crude protein (14.30%), crude fat (20.57%), ash (2.90%), fiber (2.44%), and carbohydrates (51.29%) [29]. They are also rich in iron (371.50 mg/100 g), magnesium (205.10 mg/100 g), vitamin C (88 mg/100 g), and B-complex vitamins [30]. Seed proteins exhibit a balanced amino acid profile, dominated by lysine, glutamic acid, and aspartic acid [31]. However, their application is constrained by antinutritional factors, including cyanide, saponins, and tannins [26], while the bioactive constituents of the seeds remain inadequately characterized due to limited GC-MS and HPLC-based qualitative and quantitative analyses [29,31].

In contrast, the pulp contains lower levels of protein and fat [32] but is rich in vitamin C, essential amino acids, calcium, and iron [33,34,35]. The pulp contains a wide range of phenolic compounds, including chlorogenic acid, rutin, and kaempferol, which contribute to its antioxidant potential (ABTS: 11.80 μmol/g) [34,35]. Nevertheless, its nutritional significance is constrained by low protein content (2.86%) and weak DPPH radical scavenging activity, which may reduce mineral bioavailability [32,34,35]. Leaves show the strongest antioxidant properties and are rich in essential micronutrients, particularly calcium (239.65 mg/100 g), vitamin B12 (5.35 mg/100 g), iron (30.30 mg/100 g), zinc (3.88 mg/100 g), and copper (1.12 mg/100 g) [36,37,38]. They also contain vitamins C, A, and E, as well as folic acid and cobalamin, along with amino acids mainly represented by glutamic and aspartic acids [38,39]. Despite these advantages, their high moisture content (91.9%) and high bitterness may restrict direct consumption [36].

Whole fruits provide a pronounced phytochemical spectrum, including momordicosides, carotenoids, and divers fatty acids [40,41,42]. The whole fruit provides a balanced composition of carbohydrates (57.56 g/100 g), protein (20.36 g/100 g), and fiber (8.41 g/100 g) [40], along with substantial amounts of calcium (612.42 mg/100 g) and potassium (413.02 mg/100 g). It also contains vitamins such as α-tocopherol (42.93 µg/g) and vitamin C (122.7 ppm) [41,42], and essential amino acids predominantly glutamic acid (124 mg/g) and lysine (98.7 mg/g) [43]. The calcium concentration in whole fruits (612.42 mg/100 g) and leaves (239.64 mg/100 g) can provide a meaningful proportion of the recommended daily calcium intake for adults, thereby supporting their role as mineral rich dietary ingredients [44]. Likewise, the reported levels of vitamin C and potassium compare favorably with those of several widely consumed fruits and vegetables, reinforcing the nutritional relevance of *M. charantia* in promoting antioxidant defense and electrolyte balance [45]. In addition, the protein content of seeds (14.30%) is notably higher than that of most edible fruits, highlighting their potential application in the development of nutrient-dense functional foods and plant-based nutraceutical products [29]. Overall, seeds appear most suitable for protein and mineral enriched nutraceuticals, leaves for antioxidant-based pharmaceutical applications, pulp for functional food formulations, and whole fruit for industrial extraction of bioactive compounds.

**Table 1:** Nutritional composition and phytochemical composition of different parts of *M. charantia*.

Plant Parts	Proximate Composition [%]	Minerals [mg/100 g]	Vitamins [mg/100 g]	Amino Acids [mg/g]	Antioxidant Activity	Bioactive Compounds	References
Seed	Moisture content: 8.50; ash content: 2.90; crude protein: 14.30; crude fiber: 2.44; crude fat: 20.57; carbohydrate content: 51.29	Iron: 371.50; magnesium: 205.10; phosphorus: 17.10; sodium: 11.42; calcium: 8.90; potassium: 5.27	Vitamin C: 88; thiamine: 0.181, riboflavin: 0.326; niacin: 1.110; pantothenic acid: 0.063; vitamin B6: 0.806; folate: 128	Cystine: 22.3; aspartic acid: 93.8; threonine: 25.2; serine: 55.0; glutamic acid: 96.0; proline: 54.4; glycine: 44.9; alanine: 51.2; valine: 42.2; isoleucine: 30.8; leucine: 64.9; tyrosine: 59.4; phenylalanine: 40.2; methionine: 27.6; histidine: 72.8; lysine: 101; arginine: 45.6	DPPH: 60.45%, FRAP: 113.85 µg/g, TPC: 17–29 µg/g	Alkaloids, flavonoids, tannins, saponins, cyanide	[25,29,30,31]
Pulp	Moisture: 7.10; ash: 4.45; fat: 1.34; fiber: 3.86; protein: 2.86	Calcium: 74.90; magnesium: 52.59; iron: 30.30; zinc: 3.88; copper: 1.12; manganese: 0.95	Vitamin C: 1.25	Asparagine: 8.6; threonine: 3.8; serine: 5.3; proline: 6.8; glycine: 9.1; alanine: 9.9; valine: 7.3; methionine: 0.8; isoleucine: 4.8; leucine: 7.6; tryptophan: 1.8; phenylalanine: 4.1; histidine: 2.3; lysine: 5.8; arginine: 4.2	TPC: 21.04 µg/g, TFC: 150.94; TTC: 1.45 mg/g, DPPH: 0.75%, ABTS: 11.80 µmol/g, FRAP: 10.84 µmol/g, polyphenols: 1.91 g/100 g, flavonoids: 20.75 mg/100 g	Oxalic acid, succinic acid, malic acid, citric acids, β-carotene, catechin, chlorogenic acid, p-coumaric acid, epicatechin, ferulic acid, kaempferol, naringenin, caffeic acid, gallic acid, rutin, chrysin, apigenin	[32,33,34,35]
Leaves	Crude protein: 2.13; crude fat: 0.61; ash content: 0.89; moisture content: 91.9; carbohydrate: 7.40	Calcium: 239.65; sodium: 40.40; potassium: 67.22; iron: 4.98; zinc: 24.5; copper: 4.96	Vitamin C: 1.35; vitamin A: 0.18; vitamin E: 0.18; vitamin B12: 5.35; folic acid: 20.60	Arginine: 58.5; lysine: 53.0; valine: 49.9; phenylalanine: 43.4; isoleucine: 39.9; threonine: 29.4; histidine: 21.1; tyrosine: 20.0; methionine: 11.7; glycine: 40.4; alanine: 39.4; serine: 31.8; proline: 30.4; cysteine: 9.1	FRAP: 433 µmol/g, TPC: 474 mg GAE/g, DPPH: 9.72 mg/mL	Gallic acid, tannic acid, (+)-catechin, caffeic acid, p-coumaric acid, benzoic acid, polyphenols, flavonoids, alkaloids	[33,36,37,38,39,40]
Fruit	Moisture: 3.88; ash: 5.22; crude protein: 20.36; crude fiber: 8.41; carbohydrate: 57.56	Folate: 0.0724; sodium: 521.71; calcium: 612.42; potassium: 413.02; magnesium: 421.76; phosphorus: 421.73; manganese: 4.61	α-tocopherol: 0.42, Vitamin C: 1.22	Cystine: 16.5; aspartic acid: 78.0; threonine: 17.4; serine: 43.5; glutamic acid: 124; proline: 49.7; glycine: 39.9; alanine: 46.7; valine: 36.7; isoleucine: 30.7; leucine: 60.5; tyrosine: 44.7; phenylalanine: 34.5; methionine: 23.6; histidine: 40.9; lysine: 98.7; arginine: 80.8	FRAP: 9.41 µmol/g, TPC: 224 mg GAE/g, DPPH: 27.6 mg/mL	Lauric acid, myristic acid, pentadecylic acid, palmitoleic acid, palmitic acid, margaric acid, linoleic acid, linolenic acid, oleic acid, stearic acid, nonadecylic acid, arachidic acid, erucic acid, behenic acid, tricosylic acid, lignoceric acid, saturated fatty acids, monounsaturated fatty acids, polyunsaturated fatty acids, total lipids, momordicosides A, momordicosides L, momordicosides K, 3β,7β,25-trihydroxycucurbita-5,23(E)-dien-19-al, momordicine I, gallic acid, tannic acid, (+)-catechin, caffeic acid (1.62 mg/L), p-coumaric,, oxalate, saponins, alkaloids, phylate, tannin, cyanogenic glycosides, neoxanthin, violaxanthin, lutein, zeaxanthin, α-carotene, β-carotene, total carotenoids	[41,42,43,46,47,48]

Abbreviations used: ABTS, 2,2′-azino-bis(3-ethylbenzothiazoline-6-sulfonic acid); DPPH, 2,2-diphenyl-1-picrylhydrazyl; FRAP, ferric reducing antioxidant power; TFC, total flavonoid content; TPC, total phenolic content; TTC, total tannin content.

## 4. Phytochemistry

Different parts of *M. charantia* show distinct antioxidant capacities. The seeds exhibit notable free radical–scavenging activity, with DPPH inhibition of 60.45%, ferric reducing antioxidant power (FRAP) of 113.85 µg/g, and total phenolic content (TPC) of 17–29 µg/g [46,47]. In comparison, the pulp shows lower DPPH activity (0.75%) but still demonstrates antioxidant potential through TPC (21.04 µg/g), ABTS (11.80 µmol/g), and FRAP (10.84 µmol/g) [34]. Among all plant parts, the leaves display the strongest antioxidant capacity, with very high FRAP (433 µmol/g) and TPC (474 mg GAE/g) [39]. The fruit is also an important dietary antioxidant source, with TPC of 224 mg GAE/g, FRAP of 9.41 µmol/g, and DPPH activity of 27.6 mg/mL [47]. Seeds contain major phytochemicals such as alkaloids (14.41 mg/100 g), flavonoids (12.09 mg/100 g), tannins (6.20 mg/100 g), and saponins (3.42 mg/100 g) [29], which contribute to strong radical-scavenging activity. The pulp is rich in organic acids, including malic acid (800.29 mg/kg) and oxalic acid (255.47 mg/kg), as well as polyphenols (1.91 g/100 g) and bioactive compounds such as β-carotene, naringenin, catechin, ferulic acid, and chlorogenic acid [32,34,35], supporting its antioxidant and metabolic effects.

**Figure 2:**
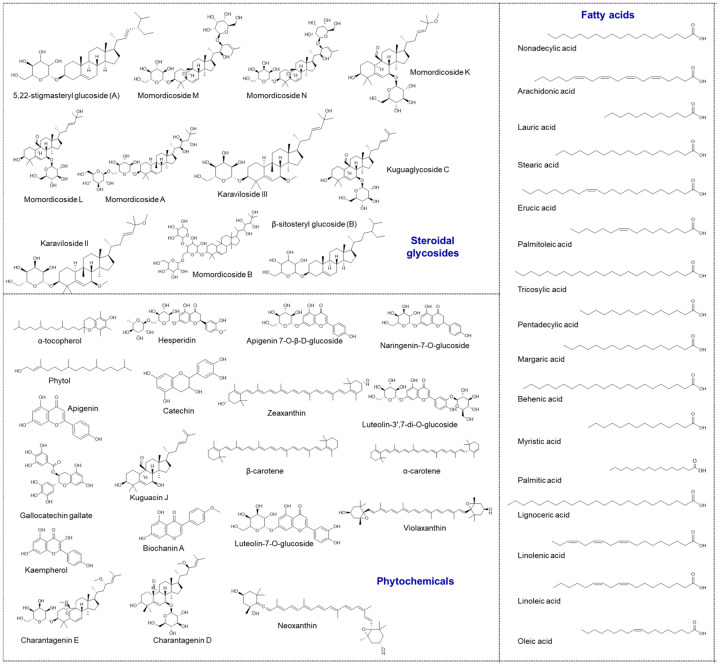
Chemical structures of phytochemicals, steroidal glycosides, and fatty acids present in seeds, pulp, juice, and leaves of *M. charantia* [29,32,34,36,40,41,42,47,48].

The leaves are particularly rich in phenolic acids such as gallic acid (95.8 mg/L) and caffeic acid (7.77 mg/L), along with flavonoids and alkaloids [36,47], which contribute to their high antioxidant activity. The fruit contains a diverse range of bioactive compounds, including carotenoids (β-carotene 42.74 µg/g; lutein 193.33 µg/g), essential fatty acids (PUFA 54.64%), and pharmacologically active cucurbitane-type triterpenoids such as momordicosides and momordicine I [41]. In addition, phenolic compounds (e.g., gallic acid 202 mg/L) and secondary metabolites such as saponins and tannins further contribute to its therapeutic potential [40,47]. It is generally suggested that the antioxidant activity follows the order leaves > seeds > fruit > pulp, but this ranking should be taken with caution. The number and interactions of bioactive compounds, extraction solvents, sample preparation methods, and analytical techniques used (such as DPPH, ABTS, and FRAP assays) have significant effects on antioxidant capacity [34,35,42,47]. Moreover, the observed data were collected from different independent studies with varying experimental conditions, rather than from a direct comparison of all plant parts. The chemical structures of phytochemicals, steroidal glycosides, and fatty acids present in the seeds, pulp, juice, and leaves of *M. charantia* are shown in Figure 2.

## 5. Advance Extraction Techniques to Improve Yield of Bioactive Compounds

Modern extraction methods for *M. charantia* have proven to be more efficient than conventional techniques, providing higher yields and recovery of bioactive compounds while significantly reducing extraction time. Among these methods, ultrasound-assisted extraction (UAE) is one of the fastest and most efficient, typically requiring only 5–30 min under optimized conditions (150–300 W ultrasound power, 30–90% ethanol). UAE yields 33.42–37.72% extract, with strong antioxidant activity (TPC 18.73 mg GAE/g; DPPH inhibition 66.93%) and high charantin content (28.56 mg/g extract) [49,50]. Optimization studies have reported even higher extract quality, with TPC of 847.91 mg GAE/100 g, FRAP of 148.76 mg AAE/g extract, and moderate antioxidant activity (DPPH IC50 119.08 µg/mL) in approximately 13 min [51]. Similarly, UAE using water as a solvent produced high polyphenol yields (104.5 mg GAE/g) and antioxidant activity (69.9%) within 12 min [52]. These findings highlight UAE as a rapid and efficient green extraction method that preserves thermolabile bioactive compounds and improves extract quality. In contrast, conventional methods such as hot reflux and Soxhlet extraction are more time- and energy-intensive, requiring longer extraction periods (about 6 h at 150 °C for hot reflux and 150 min for Soxhlet) with lower or inconsistent recovery of bioactive compounds [51,53]. Although Soxhlet extraction can produce a high overall yield (97.51%), the concentration of bioactives, including charantin, remains relatively low (0.24 mg/g), possibly due to thermal degradation or non-selective extraction. Pressurized liquid extraction (PLE) achieves similarly high yields (96.05%) under controlled conditions of high temperature (120 °C) and pressure (10 MPa), but charantin recovery is moderate (0.126 mg/g) [54]. Green technologies such as supercritical fluid extraction (SFE) with CO_2_ offer high selectivity and purity but relatively low yields (1.924%), despite increased charantin content (0.7817 mg/g) [54].

In addition to extraction efficiency, several economic and operational factors must be considered for the practical application of these extraction technologies. UAE is generally associated with moderate equipment costs, low energy consumption, short processing times, and relatively simple operation, making it attractive for industrial-scale production of food and nutraceutical ingredients [50]. However, conventional extraction techniques like Soxhlet and hot reflux extraction are more energy-intensive, time-consuming, and solvent-intensive, resulting in significant operational costs and environmental impact [53,54]. PLE has significantly higher extraction yields but involves more capital investment and technical expertise to operate the high-pressure equipment [54]. Similarly, SFE provides solvent-free extracts and excellent selectivity; however, its widespread application is often limited by high equipment costs, complex operation, and stringent process control requirements [55]. In terms of regulatory acceptability, processes that use food-grade solvents like water and ethanol are generally preferred over those that use organic solvents like acetone or dichloromethane for food, nutraceutical and pharmaceutical applications [56]. EAE and MAE are two alternative methods that have been found to significantly improve extraction efficiency, reduce solvent use and enhance the recovery of bioactive compounds [16,17], but no study has been found that reported the use of EAE and MAE for the extraction of bioactive constituents from *M. charantia*. Hence, further research on these methods is recommended as a viable approach for sustainable and scalable extraction of *M. charantia* extracts. Overall, although PLE and SFE have been shown to extract compounds more effectively, UAE appears to be the most promising method for the large-scale utilization of *M. charantia* bioactives in terms of extraction efficiency, cost-effectiveness, scalability, environmental compatibility, and regulatory compliance [51]. The impact of different green extraction techniques on the yield of bioactive compounds is summarized in Table 2.

## 6. Therapeutic Potential and Molecular Mechanism of *M. charantia* Activity

### 6.1. Anticancer Mechanisms

Bioactive compounds from *M. charantia*, particularly charantin, MAP30, and α-momorcharin, exhibit anticancer activity by inducing apoptosis through the activation of caspases-3, -8, and -9, altering mitochondrial membrane potential, and regulating pro- and anti-apoptotic proteins [60]. They also inhibit cell proliferation by causing cell-cycle arrest at the G_0_/G_1_ or S phase through downregulation of cyclins (Cyclin D, Cyclin A) and cyclin-dependent kinases, while suppressing oncogenic signaling pathways such as PI3K/Akt/mTOR and MAPK [61].

Previously published review papers on bitter melon extracts have shown broad anticancer activity across various cancer types by inducing apoptosis, inhibiting cell proliferation and angiogenesis, and modulating cancer-related signaling pathways [20,61]. These effects are largely attributed to its diverse bioactive compounds, particularly triterpenoids, peptides, and phenolic acids, which suppress tumor growth and metastasis [62]. These compounds further disrupt cancer cell metabolism, including glycolysis and lipogenesis, induce autophagy, and inhibit tumor progression, metastasis-associated proteins and key transcription factors, resulting in multi-targeted anticancer effects [63].

At the cellular and molecular levels, recent studies have identified several gene targets and signaling pathways involved in the anticancer effects of *M. charantia*. MAP30, a ribosome-inactivating protein, suppresses bladder cancer progression by inhibiting centromere protein A (CENPA), thereby reducing proliferation, promoting apoptosis, and limiting tumor growth [62]. Similarly, α-momorcharin (α-MMC) induces cell death in lung cancer cells by activating the TNF-mediated caspase cascade, inhibiting NF-κB and MAPK signaling, and causing cell-cycle arrest at the G_0_/G_1_ and S phases [63]. Extracellular vesicle-like particles derived from *M. charantia* trigger both apoptosis and ferroptosis in cervical cancer cells by blocking the Bcl-2/Bax/Akt signaling axis and inducing iron-dependent lipid peroxidation through GPX4 inhibition, representing a regulated form of cell death [64].

Another important mechanism involves metabolic reprogramming. Momordicine-I suppresses glycolysis and lipogenesis in head and neck cancer by downregulating key metabolic enzymes (GLUT1, HK1, FASN), activating the AMPK pathway, and inhibiting mTOR signaling [65]. These metabolic disruptions counter the Warburg effect, depriving tumor cells of energy and promoting apoptosis. *M. charantia* extracts also inhibit melanoma growth by downregulating PAX3 and the PI3K/Akt/mTOR pathway, thereby reducing tumor invasiveness and metastasis [66].

Beyond its direct anticancer effects, bitter melon may act synergistically with conventional therapies. Co-administration of *M. charantia* extract with rosuvastatin enhanced anticancer activity against HepG2 liver cancer cells by promoting apoptosis, increasing oxidative stress, disrupting cholesterol metabolism, and modulating drug pharmacokinetics [67]. In prostate cancer cells, *M. charantia* and other medicinal plants show significant cytotoxicity through reactive oxygen species generation, androgen receptor inhibition, apoptosis induction, and cell-cycle arrest [66]. Moreover, *M. charantia* functions as a multifunctional anticancer agent targeting several cancer hallmarks, including metabolic reprogramming, oxidative stress, apoptosis, ferroptosis, and oncogenic signaling pathways [63,64,65,66]. Its ability to modulate diverse molecular targets, together with advances in delivery systems, highlights its translational potential for future cancer therapies. However, further clinical trials and genetic validation studies are needed to confirm its efficacy and safety in humans, despite promising preclinical evidence.

#### 6.1.1. Liver Cancer

*M. charantia* exhibits anticancer activity against hepatocellular carcinoma (HCC) through multiple mechanisms, including apoptosis induction, metabolic disruption, anti-inflammatory effects, and suppression of carcinogenic processes [68]. A key mechanism is the activation of mitochondria-mediated apoptosis, where bioactive compounds such as MAP30 and α-momorcharin regulate caspase cascades, alter mitochondrial membrane potential, and trigger intrinsic apoptotic signaling in liver cancer cells [69]. Ribonuclease MC2 also shows antitumor activity by degrading RNA, inhibiting protein synthesis, and inducing caspase-dependent apoptosis in hepatoma models both in vitro and in vivo [70].

Bioactive compounds of *M. charantia* modulate key oncogenic signaling pathways. Cucurbitane-type triterpenoids exhibit cytotoxic effects by regulating cellular pathways that control cell growth and survival, thereby inhibiting hepatoma cell proliferation [71,72]. Bitter melon also suppresses inflammatory and metabolic signaling pathways such as NF-κB and JNK, which are closely associated with hepatocarcinogenesis, reducing tumor-promoting inflammation and increasing oxidative stress and thereby contributing to hepatoma cell death [73]. Bitter melon extracts further regulate lipid metabolism and lipid peroxidation in chemically induced liver cancer models, suggesting a role in controlling oxidative damage and tumor development [74]. These effects support the chemopreventive potential of *M. charantia*, as it modulates several hallmarks of hepatocarcinogenesis, including proliferation, inflammation, and genomic instability [75]. Additionally, its antiviral activity against hepatitis B virus may indirectly reduce liver cancer risk by limiting virus-associated carcinogenesis [76]. However, α-momorcharin (α-MMC) can also exhibit cytotoxic effects on normal liver cells through activation of the JNK pathway, indicating a narrow therapeutic index and the need for controlled dosing strategies [77,78]. Overall, *M. charantia* represents a promising candidate for liver cancer prevention and therapeutic strategies due to its combined effects on apoptosis induction, metabolic reprogramming, anti-inflammatory signaling, and chemoprevention.

#### 6.1.2. Colorectal Cancer

*M. charantia* has shown anticancer potential against colorectal cancer through multiple interconnected mechanisms, including apoptosis induction, anti-inflammatory effects, and chemoprevention. BG-4 significantly suppresses human colon cancer cell growth by inducing cell death through activation of the caspase pathway and disruption of cellular homeostasis [21]. Bitter gourd extracts also trigger mitochondria-dependent apoptosis, characterized by loss of mitochondrial membrane potential, cytochrome c release, and activation of caspase-3 and caspase-9, leading to programmed cancer cell death [79].

Network pharmacology analyses indicate that various *M. charantia* phytochemicals interact with key proteins and signaling pathways involved in cancer, supporting a multi-targeted mechanism that regulates cell proliferation, apoptosis, and survival pathways during colorectal tumor progression [80]. Bitter gourd also exerts anti-inflammatory and cytotoxic effects by inhibiting upstream regulators such as TAK1, thereby suppressing pro-inflammatory cascades associated with colorectal carcinogenesis [81]. In addition, its anticlastogenic activity helps prevent DNA damage and chromosomal abnormalities, reducing mutation rates and cancer development [75]. In this regard, *M. charantia* targets several hallmarks of colorectal cancer, including uncontrolled proliferation, resistance to apoptosis, chronic inflammation, and genomic instability, highlighting its potential as a chemopreventive and therapeutic agent.

#### 6.1.3. Breast Cancer

*M. charantia* displays activity against breast cancer through a multi-targeted approach affecting cell proliferation, apoptosis, metabolism, and lipid signaling. Bitter melon extracts suppress breast cancer cell growth by regulating cell-cycle genes, leading to cell-cycle arrest and apoptosis through caspase-dependent pathways [82]. They can also induce lethal oxidative stress in carcinogenic breast cells by disrupting cellular energy metabolism and increasing reactive oxygen species (ROS) production [83].

At the molecular level, ribonucleases such as RNase MC2 and ribosome-inactivating proteins (e.g., α-momorcharin) contribute to anticancer activity by inhibiting protein synthesis, activating MAPK signaling, and inducing caspase-dependent apoptosis [67,84]. Cucurbitane-type triterpenoids further promote apoptosis and autophagy through activation of peroxisome proliferator-activated receptor gamma (PPAR-γ), thereby inhibiting tumor growth [85,86]. Another tumor-suppressive mechanism disrupts cholesterol esterification, which is crucial for the rapid proliferation of triple-negative breast cancer cells [87].

**Figure 3:**
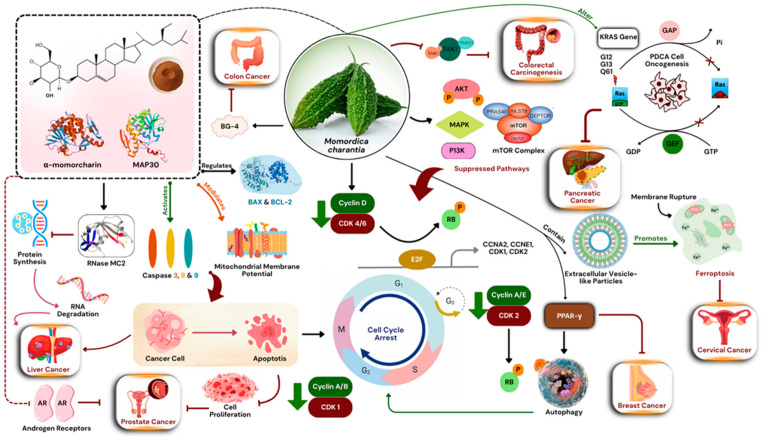
Anticancer properties of bioactive compounds including charantin, α-momorcharin, and MAP30 (Momordica anti-HIV protein of 30 kDa) of *M. charantia* through multiple molecular mechanisms. These compounds inhibit protein synthesis and induce RNA degradation through *M. charantia* ribonuclease (RNase MC2) activity, suppressing cancer cells in liver cancer. They activate apoptosis by increasing BAX (BCL-2-Associated X Protein) and decreasing BCL-2 (B-cell lymphoma 2), disrupting mitochondrial membrane potential and activating caspase-3, caspase-8, and caspase-9, resulting in programmed cancer cell death. *M. charantia* induces cell-cycle arrest by downregulating Cyclin D, Cyclin A/E, Cyclin A/B, CDK1, CDK2, and CDK4/6 (cyclin-dependent kinases), preventing RB (retinoblastoma protein) phosphorylation and suppressing E2F transcription factor-mediated cell proliferation. It also inhibits major oncogenic signaling pathways including PI3K (phosphoinositide 3-kinase), AKT (protein kinase B), MAPK (mitogen-activated protein kinase), and mTOR (mammalian target of rapamycin), reducing tumor growth and survival in pancreatic cancer. They suppress KRAS (Kirsten rat sarcoma viral oncogene homolog)-mediated oncogenesis by interfering with GDP (guanosine diphosphate)/GTP (guanosine triphosphate) exchange regulated by GEF (guanine nucleotide exchange factor) and GAP (GTPase-activating protein), inhibiting pancreatic ductal adenocarcinoma progression. Inhibition of TAK1 (transforming growth factor-β activated kinase 1) and TAB1/2/3 (TAK1-binding proteins 1, 2, and 3) downregulate colorectal carcinogenesis. Extracellular vesicle-like particles promote ferroptosis through ROS (reactive oxygen species) accumulation, Fe^2+^-dependent lipid peroxidation, and membrane rupture in cervical cancer and activation of PPAR-γ (peroxisome proliferator-activated receptor gamma) stimulates autophagy, inhibit breast cancer [60,63,64,65,66,67,79,84,85,86,87].

Bitter melon also shows chemopreventive and adjuvant therapeutic potential. Its phytochemicals modulate oncogenic pathways involved in proliferation, apoptosis, and inflammation, supporting its role as a multi-target anticancer agent [88,89]. Extracellular vesicles derived from bitter melon enhance anticancer effects by delivering bioactive molecules that inhibit tumor proliferation and survival signaling [90]. Additionally, *M. charantia* may improve therapeutic efficacy by increasing the intracellular accumulation of drugs such as paclitaxel in breast cancer cells [91]. In animal models, it also regulates lipid metabolism, improving lipid profiles and reducing the risk of mammary tumors [92,93]. However, α-momorcharin has a relatively narrow therapeutic index, and strategies such as pegylation may help reduce toxicity while maintaining efficacy [94,95]. Overall, *M. charantia* represents a promising candidate for breast cancer prevention and adjunct therapy by targeting key cancer hallmarks, including uncontrolled proliferation, metabolic reprogramming, resistance to cell death, and altered lipid metabolism. The anticancer properties of bioactive compounds, including charantin, α-momorcharin, and MAP30 (Momordica anti-HIV protein of 30 kDa) from *M. charantia,* through multiple molecular mechanisms in different cancers, are shown in Figure 3.

#### 6.1.4. Lung Cancer

*M. charantia* shows significant anticancer activity against human A549 lung cancer cells through multiple mechanisms. A major pathway involves apoptosis induced by ROS, which cause mitochondrial dysfunction, loss of mitochondrial membrane potential, and activation of apoptotic signaling pathways [83,96]. Oxidative stress damages cellular components and activates caspase-dependent cascades that promote programmed cell death [97]. Ribosome-inactivating proteins such as α-momorcharin (α-MMC) and MAP30 further enhance apoptosis and cell-cycle arrest through caspase activation and tumor necrosis factor (TNF)–related signaling pathways [98,99]. In addition to apoptotic mechanisms, *M. charantia* can induce non-apoptotic cell death by disrupting cellular energy metabolism, impairing mitochondrial respiration and ATP synthesis, and increasing oxidative stress [83]. This dual action highlights its potential to target cancer cells that develop resistance to apoptosis-based therapies.

*M. charantia* extracts also demonstrate anti-migratory and anti-invasive effects in lung cancer cells, suggesting a metastasis-preventive role through modulation of pathways involved in cytoskeletal organization, cell adhesion, and extracellular matrix degradation [99,100]. In summary, the anticancer effects of *M. charantia* in lung cancer involve ROS-mediated oxidative stress, mitochondrial damage, caspase-dependent apoptosis, metabolic disruption, and inhibition of tumor progression and metastasis. Despite promising experimental evidence, further in vivo and clinical studies are required to confirm its efficacy and safety in lung cancer prevention and therapy.

#### 6.1.5. Pancreatic Cancer

*M. charantia* shows strong anticancer potential against pancreatic cancer through multiple complementary molecular mechanisms. Extracts and juice of *M. charantia* demonstrate significant anti-tumor activity across different cultivars, suggesting similar bioactivity regardless of geographic origin [101]. A key mechanism involves targeting both bulk tumor cells and pancreatic cancer stem cells, thereby reducing tumor formation, progression, and recurrence [102]. Cancer stem cells are major contributors to therapy resistance and disease relapse. *M. charantia* also suppresses oncogenic signaling pathways, including KRAS mutations that play a central role in pancreatic tumorigenesis, indicating its ability to interfere with critical genetic drivers of cancer development [103]. Its anticancer effects include induction of apoptosis, inhibition of cell proliferation, and disruption of metabolic pathways required for cancer cell survival. Cucurbitane-type triterpenoids contribute to these cytotoxic effects and may also provide protection against oxidative stress in pancreatic cells [72]. In addition, bitter melon can help overcome chemoresistance to gemcitabine, a standard chemotherapeutic drug for pancreatic cancer, by modulating drug-resistance pathways, suppressing survival signaling, and increasing drug sensitivity [104,105]. This suggests potential use as an adjuvant therapy to enhance chemotherapy efficacy and that *M. charantia* exerts multifaceted anticancer effects in pancreatic cancer, including tumor growth inhibition, targeting of cancer stem cells, modulation of KRAS-related pathways, induction of apoptosis, metabolic disruption, and reversal of chemoresistance [23]. Although promising, further clinical studies are needed to confirm its efficacy and safety in humans. The anticancer effects of *M. charantia* against specific cancer cell lines and their underlying molecular mechanisms are summarized in Table 3.

### 6.2. Hepatoprotective Mechanisms

The hepatoprotective effects of *M. charantia* are mainly mediated through antioxidant activity, improved hepatic lipid metabolism, and regulation of inflammatory pathways [113]. Bitter melon bioactives enhance endogenous antioxidant defenses by increasing superoxide dismutase (SOD), catalase (CAT), and glutathione (GSH) levels, thereby reducing lipid peroxidation (MDA) and protecting hepatocyte integrity [114]. They also regulate hepatic lipid homeostasis by suppressing de novo lipogenesis through down regulation of SREBP-1c, ACC, and FASN, while promoting β-oxidation via AMPK activation [115].

These compounds further inhibit hepatic inflammation by blocking NF-κB signaling and reducing pro-inflammatory cytokines including TNF-α, IL-6. Consequently, hepatic injury is attenuated, leading to improvements in liver function markers such as ALT, AST, ALP [116,117]. Beyond their anti-inflammatory effects, cucurbitane-type triterpenoids help to prevent hepatic fibrosis by modulating TGF signaling pathways, thereby limiting extracellular matrix deposition and fibrotic progression [72,115]. The anti-fibrotic activity of these compounds may also contribute to the prevention of hepatocarcinogenesis. Furthermore, activation of metabolic regulators including PPAR-α, AMPK, and SIRT1, enhances fatty acid oxidation, improves mitochondrial function, and restores cellular energy balance in the liver [115]. These metabolic adaptations reduce hepatic lipid accumulation and slow the progression of fatty liver disease, thereby reinforcing the overall hepatoprotective effect of *M. charantia* [85,116].

**Figure 4:**
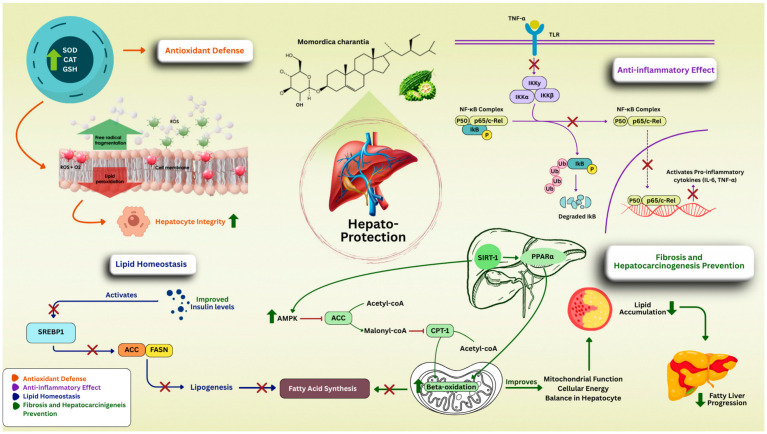
Hepatoprotective mechanisms of *M. charantia* involve interconnected antioxidant, anti-inflammatory, lipid-regulating, and mitochondrial protective effects. The plant enhances endogenous antioxidant enzymes—superoxide dismutase (SOD), catalase (CAT), and reduced glutathione (GSH)—which scavenge reactive oxygen species (ROS), reduce lipid peroxidation, and protect hepatocyte membrane integrity. *M. charantia* regulates lipid metabolism primarily through activation of AMP-activated protein kinase (AMPK). AMPK inhibits acetyl-CoA carboxylase (ACC), reducing conversion of acetyl-CoA to malonyl-CoA and suppressing fatty acid synthesis, while upregulating carnitine palmitoyltransferase-1 (CPT-1)–mediated β-oxidation in mitochondria. This improves mitochondrial function, maintains cellular energy balance, and reduces hepatic lipid accumulation. It also suppresses lipogenic regulators such as sterol regulatory element-binding protein-1 (SREBP-1), fatty acid synthase (FASN), mechanistic target of rapamycin complex 1 (mTORC1), carbohydrate-responsive element-binding protein (ChREBP), and citrate carrier (CiC), thereby limiting lipogenesis. Increased glucose transporter-5 (GLUT5) activity and modulation of insulin and ammonia metabolism further support metabolic homeostasis. Additionally, *M. charantia* activates sirtuin-1 (SIRT1) and peroxisome proliferator-activated receptor-α (PPAR-α), promoting β-oxidation and preventing fatty liver progression. Its anti-inflammatory effects involve inhibition of toll-like receptor (TLR)–mediated nuclear factor kappa-B (NF-κB) signaling by suppressing inhibitor of NF-κB kinase subunits (IKKα, IKKβ, IKKγ), preventing degradation of inhibitor of κB (IκB), and blocking activation of NF-κB p50/p65 (c-Rel) complexes. Bitter melon also inhibits pro-inflammatory signaling pathways such as NF-κB and JNK, which reduces cytokine-mediated liver injury and fibrosis [5,72,82,113,114,115,116,117,118,119,120,121,122].

Charantin-rich extracts exhibit strong antioxidant activity, reducing oxidative stress and lipid peroxidation and thereby protecting hepatocytes from chemical and carcinogenic injury [117,118]. Bitter melon supplementation has also been shown to slow fibrosis progression, oxidative stress, and microenvironmental alterations in chemically induced liver injury and carcinogenesis models, indicating its potential to prevent liver damage and inhibit hepatocellular carcinoma development [92,115]. In hepatotoxicity models, *M. charantia* suppresses apoptosis-related signaling, including caspase-9 activation, and decreases inflammatory cytokines such as IL-1β, promoting liver regeneration [119]. Charantin further regulates lipid metabolism, reducing hepatic steatosis by improving cholesterol and triglyceride homeostasis and limiting fatty acid accumulation in the liver [120,121]. Bitter melon supplementation has also been shown to slow fibrosis progression, oxidative stress, and microenvironmental alterations in chemically induced liver injury and carcinogenesis models, indicating its potential to prevent liver damage and inhibit hepatocellular carcinoma development [115,117]. Additionally, it protects against alcohol- and toxin-induced liver injury by counteracting oxidative stress and inflammatory processes [122]. Therefore, charantin and related cucurbitane-type compounds act through multiple mechanisms, including enhanced antioxidant defense, inhibition of inflammatory signaling, regulation of lipid metabolism, and anti-fibrotic activity, making them promising natural agents for liver protection and prevention of liver disease progression. The hepatoprotective mechanisms of bioactive compounds of *M. charantia* are illustrated in Figure 4.

### 6.3. Antimicrobial Mechanisms

The antimicrobial activity of *M. charantia* is mainly mediated through disruption of microbial cell membranes, leading to leakage of cellular contents and inhibition of microbial growth [123]. Bioactive compounds interact with membrane lipids and proteins, causing structural damage and increased permeability. They also inhibit nucleic acid and protein synthesis as well as essential enzymatic activities in bacteria and fungi. In addition, these compounds interfere with quorum sensing, reducing biofilm formation and virulence in a broad range of Gram-positive and Gram-negative bacteria and some fungal pathogens [124]. Cucurbitane-type triterpenoids are key contributors to the antimicrobial activity of *M. charantia*. Charantin and other phytochemicals exhibit broad-spectrum antimicrobial effects by disrupting microbial membrane integrity, inhibiting essential metabolic pathways, and inducing oxidative stress in microbial cells [25,125]. Bitter gourd extracts can also enhance ROS generation, leading to lipid peroxidation, protein denaturation, and microbial cell death [126]. Furthermore, *M. charantia* bioactive compounds may overcome microbial resistance by inhibiting efflux pumps and increasing intracellular accumulation of antimicrobial agents [127]. Polysaccharides and proteins in the plant can further enhance antimicrobial effects by supporting immune responses and inhibiting microbial adhesion and growth [128]. Overall, charantin acts through multiple mechanisms, including membrane disruption, oxidative damage, and interference with microbial survival pathways, highlighting its potential as a natural antimicrobial agent.

### 6.4. Antidiabetic Mechanisms

The antidiabetic effects of *M. charantia* involve multiple mechanisms, including stimulation of insulin secretion, enhanced GLUT-4 translocation in muscle and adipose tissues, and improved insulin sensitivity through AMPK activation [113,116]. Bioactive compounds such as charantin inhibit hepatic gluconeogenic enzymes, reducing hepatic glucose production and regulating key enzymes involved in glycolysis and glycogenesis [129]. *M. charantia* further modulates incretin hormones such as GLP-1, delays carbohydrate absorption by inhibiting α-glucosidase and α-amylase, and reduces oxidative stress and inflammation associated with insulin resistance [130].

*M. charantia* has shown beneficial effects on glycemic control, insulin sensitivity, and metabolic homeostasis in both experimental and clinical studies. Clinical trials and meta-analyses report reductions in fasting blood glucose, HbA1c, and insulin resistance, although outcomes vary depending on dosage, preparation, and study design [129,130,131,132]. Its hypoglycemic effects involve multiple pathways, including activation of AMP-activated protein kinase (AMPK), insulin receptor substrate-1 (IRS-1), PI3K, and Akt signaling, which enhance glucose uptake and utilization, particularly through increased GLUT4 translocation in peripheral tissues [128]. *M. charantia* also improves pancreatic β-cell function by stimulating insulin secretion and protecting these cells from oxidative stress and apoptosis [133,134]. Additionally, it reduces intestinal glucose absorption by inhibiting α-glucosidase and α-amylase enzymes [135,136].

**Figure 5:**
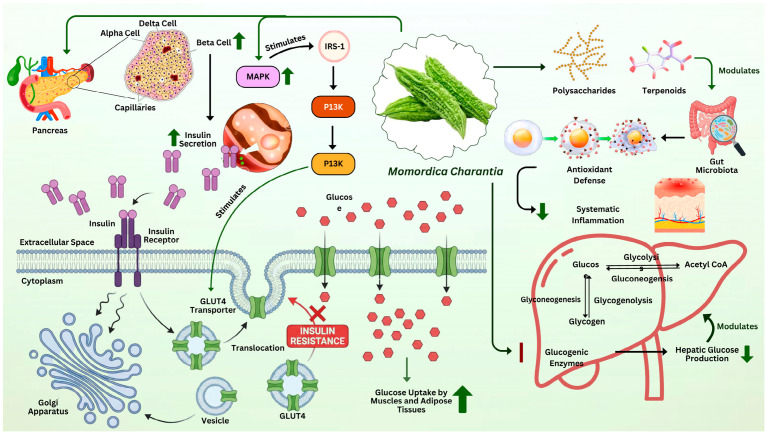
*M. charantia* exhibits antidiabetic activity through multiple mechanisms, including stimulation of insulin secretion, improved insulin signaling, enhanced glucose uptake, antioxidant defense, modulation of gut microbiota, and regulation of hepatic glucose metabolism. It stimulates pancreatic β-cells, increasing insulin secretion, while interactions among α-, β-, and δ-cells help maintain glucose homeostasis. Released insulin binds to insulin receptors on target cells and activates intracellular pathways such as mitogen-activated protein kinase (MAPK), insulin receptor substrate-1 (IRS-1), and phosphoinositide 3-kinase (PI3K). Activation of the IRS-1/PI3K pathway promotes translocation of glucose transporter type 4 (GLUT4) vesicles from the Golgi apparatus to the plasma membrane, increasing glucose uptake in muscle and adipose tissues and reducing insulin resistance. *M. charantia* also enhances antioxidant defenses through its polysaccharides and terpenoids, reducing oxidative stress and systemic inflammation, thereby improving insulin sensitivity. In addition, it modulates gut microbiota composition to support metabolic balance and anti-inflammatory responses. The plant further regulates carbohydrate metabolism by suppressing hepatic gluconeogenesis and gluconeogenic enzymes, reducing hepatic glucose production, and modulating glycolysis, glycogenolysis, glycogenesis, and acetyl-coenzyme A (acetyl-CoA) metabolism [75,115,116,128,129,130,131,132,133,134,135,136,137,138,139,140,141,142,143].

Polysaccharides and triterpenoids from the plant further modulate gut microbiota composition, decrease systemic inflammation, and enhance antioxidant defenses via the Nrf2 pathway [137,138,139]. Emerging evidence also suggests protective effects against diabetes-related complications, including retinopathy, neurodegeneration and reproductive dysfunction, through anti-inflammatory, anti-apoptotic and neuroprotective mechanisms [140,141]. However, long-term use may cause cytotoxic or reproductive effects, highlighting the need for standardized dosing and quality control [75,136]. The multi-targeted actions of *M. charantia*, including metabolic regulation, enzyme inhibition, antioxidant activity, and signaling modulation, support its potential as a complementary therapy for type 2 diabetes management [142,143]. The antidiabetic mechanisms and molecular targets of *M. charantia* are illustrated in Figure 5.

### 6.5. Antioxidant Potential

The antioxidant activity of *M. charantia* is largely attributed to its high content of phenolics, flavonoids, carotenoids, and bioactive peptides, which scavenge reactive oxygen species (ROS) and reactive nitrogen species (RNS). *M. charantia* further exerts antioxidant effects by neutralizing ROS, inhibiting lipid peroxidation, and strengthening endogenous antioxidant systems such as SOD, CAT, and GPx [144]. They also activate the Nrf2/ARE signaling pathway, promoting the expression of antioxidant and detoxifying genes and thereby reducing oxidative damage to lipids, proteins, and DNA associated with chronic diseases [139,140]. Different plant parts, including fruits, seeds, and leaves, show strong free radical scavenging and reducing capacity due to their high phenolic and phytochemical content [144]. Processing methods such as microwave heating can modify and sometimes enhance the bioavailability and activity of these antioxidant compounds by altering phenolic and carotenoid profiles [145].

Its bioactive compounds also prevent protein denaturation and oxidative damage to biomolecules, contributing to anti-inflammatory and cytoprotective effects [146,147]. Emerging approaches, including *M. charantia*-derived extracellular vesicles, can deliver antioxidant molecules and regulatory factors to target tissues, thereby reducing oxidative stress and improving intestinal health in conditions such as ulcerative colitis [148,149].

Green synthesis of zinc oxide nanoparticles using *M. charantia* extracts has also been reported to enhance antioxidant and antimicrobial properties, indicating synergistic effects between plant phytochemicals and nanomaterials [150]. Additionally, elicitation strategies, such as treatment with plant hormones or signaling compounds, can increase the accumulation of antioxidant phytochemicals, further enhancing the plant’s medicinal value [151]. Overall, the antioxidant effects of *M. charantia* involve direct radical scavenging, modulation of antioxidant enzymes, protection of biomolecules, and emerging delivery technologies that support its therapeutic and nutraceutical applications.

### 6.6. Anti-Inflammatory Mechanisms

The anti-inflammatory activity of *M. charantia* is mainly mediated through inhibition of key signaling pathways, particularly NF-κB and MAPK, resulting in reduced levels of pro-inflammatory cytokines such as TNF-α, IL-1β, and IL-6 [27,60,62,99,124]. Its bioactive compounds also suppress transcription factors involved in inflammatory gene expression and inhibit the production of inflammatory mediators, including prostaglandins and nitric oxide, by downregulating COX-2 and iNOS enzymes [78,109]. In addition, *M. charantia* promotes the production of anti-inflammatory cytokines such as IL-10, contributing to immune homeostasis. It also influences macrophage activation and polarization, thereby reducing chronic inflammation associated with metabolic and degenerative diseases [148].

*M. charantia* exhibits a broad range of pharmacological activities due to bioactive compounds such as triterpenoids, polysaccharides, peptides, and phenolics, which act through multiple interconnected molecular mechanisms [9,10]. It shows strong immunomodulatory and anti-inflammatory effects by inhibiting pro-inflammatory cytokines (TNF-α, IL-6), promoting anti-inflammatory mediators, suppressing NF-κB and MAPK signaling, and regulating macrophage polarization [148]. Its antidiabetic activity involves activation of the AMPK pathway, enhanced GLUT4 translocation, inhibition of carbohydrate-digesting enzymes, and protection of pancreatic β-cells from oxidative stress, thereby improving glucose homeostasis [109,140]. *M. charantia* also demonstrates anticancer properties by inducing caspase-mediated apoptosis, causing cell-cycle arrest, and inhibiting the PI3K/Akt pathway, particularly through cucurbitane-type triterpenoids [109].

**Figure 6:**
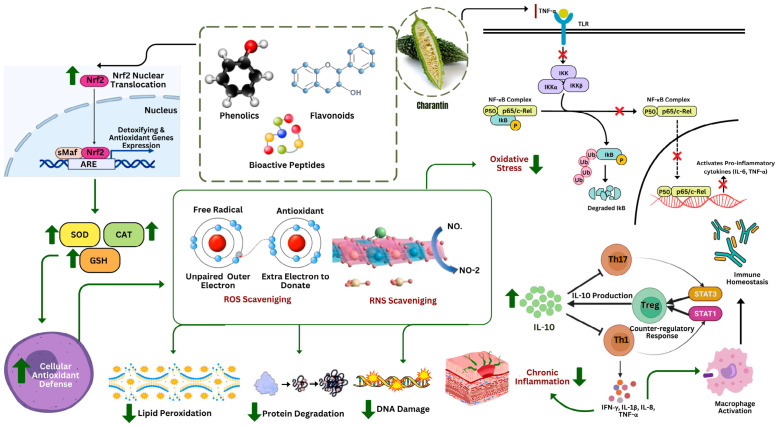
*M. charantia* exhibits antioxidant and anti-inflammatory activities through the synergistic action of phenolics, flavonoids, and bioactive peptides that scavenge reactive oxygen species (ROS) and reactive nitrogen species (RNS). These compounds donate electrons to stabilize free radicals, thereby reducing oxidative stress and strengthening cellular antioxidant defenses. They activate nuclear factor erythroid 2–related factor 2 (Nrf2), promoting its nuclear translocation and binding with small musculoaponeurotic fibrosarcoma protein (sMaf) to antioxidant response element (ARE) sequences, which stimulates the expression of detoxifying and antioxidant genes. This activation increases endogenous antioxidant enzymes, including superoxide dismutase (SOD), catalase (CAT), and reduced glutathione (GSH), thereby limiting lipid peroxidation, protein degradation, DNA damage, and overall oxidative injury. *M. charantia* also suppresses inflammatory signaling by inhibiting toll-like receptor (TLR)–mediated activation of nuclear factor kappa-B (NF-κB). It blocks inhibitor of NF-κB kinase subunits (IKKα, IKKβ, IKKγ), preventing phosphorylation and degradation of inhibitor of κB (IκB) and inhibiting translocation of NF-κB p50/p65 (c-Rel) complexes. This reduces the expression of pro-inflammatory cytokines such as tumor necrosis factor-α (TNF-α), interferon-γ (IFN-γ), interleukin-1β (IL-1β), interleukin-6 (IL-6), and interleukin-8 (IL-8). In parallel, it enhances interleukin-10 (IL-10) production and activates signal transducer and activator of transcription 1 and 3 (STAT1 and STAT3), promoting regulatory T-cell (Treg) responses while suppressing T helper 1 (Th1) and T helper 17 (Th17)–mediated inflammation. Together, these mechanisms maintain immune homeostasis, regulate macrophage activation, and reduce chronic inflammation and oxidative tissue injury [139,140,141,142,143,144,145,146,147,148,149,150,151,152].

Additionally, extracellular vesicles derived from *M. charantia* may support gastrointestinal health and treatment of ulcerative colitis by modulating gut microbiota, restoring intestinal barrier integrity, and reducing inflammation [149]. Its antioxidant activity is linked to the activation of the Nrf2/ARE pathway, which enhances endogenous antioxidant enzymes and reduces oxidative stress [147,152]. The plant also shows cardiovascular protective effects by improving nitric oxide bioavailability, reducing vascular inflammation, and regulating lipid metabolism, as well as neuroprotective effects through inhibition of β-amyloid aggregation and neuroinflammation [22,140]. In sum, the therapeutic potential of *M. charantia* arises from its multi-target actions involving metabolic regulation, immune modulation, antioxidant defense, and cellular signaling pathways, supporting its application in nutraceutical and pharmaceutical development [137,152]. The antioxidant and anti-inflammatory mechanisms and molecular targets of *M. charantia* are illustrated in Figure 6.

## 7. Industrial Applications

### 7.1. Applications in Food Industries

The food industry plays an important role in improving food security, public health, and the development of innovative products to meet the nutritional needs of growing populations. Fortification with vegetable powders or extracts is an effective strategy to enhance the nutritional value of foods by enriching them with minerals, vitamins, bioactive compounds, and dietary fiber [153]. Such ingredients also support the development of functional and value-added foods with additional health benefits.

Incorporation of bitter gourd seed powder (BGSP) into biscuits at different levels (0%, 5%, 10%, 15%, and 20%) as a substitute for refined wheat flour significantly improved protein, fiber, mineral, total phenolic, and flavonoid contents. However, BGSP reduced biscuit spread ratio, thickness, and diameter, with 5% BGSP showing the highest sensory acceptability [154].

Fortification of whole-wheat cookies with bitter gourd (BG) powder also lowered the glycemic index and improved serum glucose levels in healthy adults. BG powder (1–3% *w*/*w*) significantly increased antioxidant activity (DPPH, FRAP), total phenolic content (TPC), and inhibition of α-amylase and α-glucosidase (*p* < 0.05). Cookies containing 1% BG powder showed the best sensory acceptance [155]. Bitter melon juice (BMJ) added to yogurt (0–5%) increased acidity and fermentation time while lowering pH. Although antioxidant capacity increased, total phenolic content (TPC), total flavonoid content (TFC), and total chlorophyll levels were not significantly improved with higher BMJ levels [156]. Mixed pickles prepared from bitter gourd and bottle gourd with spices have also been reported to help improve blood glucose levels in diabetic patients [157].

Bread fortified with bitter gourd powder showed improved nutritional composition, including higher moisture, carbohydrates, protein, fat, fiber, ash, and minerals such as calcium, iron, zinc, and magnesium, with an energy value of 242.82 kcal/100 g [158]. A functional beverage containing bitter gourd, lemon, and amla was also developed and evaluated for over two months. During storage, total soluble solids, titratable acidity, and ascorbic acid decreased, while pH increased. The most acceptable formulation contained 6% bitter gourd, 4% lemon, and 3% amla, suggesting potential for commercial functional beverages [159].

Bitter gourd seed flour has also been developed with the following composition: moisture (8.50%), ash (2.90%), protein (14.30%), fiber (2.44%), fat (20.57%), and carbohydrates (51.29%). It is rich in iron (371.50 mg/100 g), magnesium (205.10 mg/100 g), and potassium (5.27 mg/100 g). Phytochemicals include alkaloids (14.41 mg/100 g), flavonoids (12.09 mg/100 g), tannins (6.20 mg/100 g), and saponins (3.42 mg/100 g), with low cyanide content (2.10 mg/100 g), indicating safety for consumption. The flour also exhibits favorable functional properties such as bulk density (0.0451), swelling capacity (103.87), water absorption (241.40), oil absorption (297.40), and variable solubility. These properties suggest that bitter gourd seed flour is a valuable nutrient source that may help address malnutrition and micronutrient deficiencies in diets [26].

### 7.2. Pharmacological Applications

The pharmacological activity of bitter gourd is largely attributed to its rich phytochemical composition, including triterpenoids, flavonoids, phenolic acids, saponins, alkaloids, charantin, and polypeptide-p. These compounds contribute to antihyperglycemic, hypolipidemic, antioxidant, anti-inflammatory, antimicrobial, anti-obesity, hepatoprotective, and anticancer effects, supporting its potential as a functional food and therapeutic agent. *M. charantia* has been widely studied for hypolipidemic and cardioprotective effects. In hypercholesterolemic Sprague–Dawley rats, different plant parts (whole fruit, seedless fruit, seeds, and seed extracts) significantly reduced serum cholesterol (18.79–40.17%), triglycerides (25.97–37.01%), and low-density lipoprotein (LDL) levels (14.49–26.09%) [160]. Bitter gourd seed oil also shows nutraceutical potential due to its high content of α-eleostearic acid and phytosterols. Soxhlet extraction yielded about 26.10% oil, and gas chromatography analysis revealed major fatty acids such as α-eleostearic (45.60%), palmitic (3.69%), stearic (28.00%), oleic (12.45%), linoleic (8.90%), arachidic (0.71%), and gadoleic acids (0.65%). This unique fatty-acid and phytosterol profile suggests antioxidant, anti-inflammatory, anti-atherosclerotic, and anti-tumor potential [161].

Bitter gourd leaves also demonstrate antioxidant and hepatoprotective effects in high-fat-diet animal models. Ethanolic leaf extracts improved serum lipid profiles by lowering total cholesterol, triglycerides, and LDL while increasing HDL levels in mice. Hepatoprotective effects were confirmed by improved liver enzyme markers and histopathological findings, indicating potential use against dyslipidemia, hepatic steatosis, and oxidative stress-related disorders [162].

Bitter gourd also exhibits insulin-mimetic properties through compounds such as charantin and polypeptide-p, which enhance glucose uptake, improve insulin sensitivity, and regulate carbohydrate metabolism. Clinical studies show that *M. charantia* capsules used alongside standard diabetes treatment can reduce glycosylated hemoglobin (HbA1c) levels in patients with poorly controlled type 2 diabetes [163]. Randomized controlled trials also reported significant reductions in fasting plasma glucose, fasting insulin, and HOMA-IR after 12 weeks of supplementation in prediabetic individuals [164].

The leaves exhibit antimicrobial activity against pathogens including *Staphylococcus aureus*, methicillin-resistant *S. aureus* (MRSA), *Salmonella typhi*, *Escherichia coli*, and *Pseudomonas aeruginosa* [165]. This activity is mainly attributed to flavonoids, tannins, terpenoids, and saponins that disrupt microbial membranes and metabolic pathways. Ethanol extracts of bitter melon leaves also showed concentration-dependent antibacterial activity against *Staphylococcus epidermidis* [166]. These findings suggest the potential use of natural antimicrobial or preservative agents in food and pharmaceutical applications.

Bitter melon bioactive compounds also exhibit anticancer effects by inhibiting tumor cell proliferation, inducing apoptosis and autophagy, suppressing angiogenesis and metastasis, modulating signaling pathways, and regulating immune responses. Notably, extracts often show selective cytotoxicity toward cancer cells with minimal toxicity to normal cells [67,68].

In addition to antidiabetic and hypolipidemic effects, bitter gourd has demonstrated anti-obesity potential. In studies using C57BL/6J mice, bitter melon seed oil fractions reduced adiposity and adipocyte size in animals fed diets containing these fractions [167]. Recent research also focuses on improving delivery systems and patient acceptability of bitter melon formulations. For example, effervescent granules prepared from *M. charantia* extracts showed favorable physicochemical properties and improved palatability, especially at higher gelatin concentrations [168]. Such formulations may enhance patient compliance and help mask the plant’s naturally bitter taste.

## 8. Limitations and Future Directions

*M. charantia* has attracted considerable interest for its nutritional, phytochemical, and therapeutic properties; however, several challenges limit its large-scale use in food and pharmaceutical industries. Significant variability in nutritional composition, phytochemical content, and bioactivity arises from differences in cultivars, geographic origin, ripeness, and extraction methods, making quality standardization and reproducible biological effects difficult. In addition, many studies focus on crude extracts without detailed characterization, purification, or quantification of active compounds, limiting understanding of mechanisms and industrial applications. Green extraction methods should be a major focus of future *M. charantia* research. Although UAE, PLE, and SFE have shown promising results [52,54,55], EAE and MAE remain largely unexplored for the recovery of *M. charantia* bioactive compounds. These methods represent promising opportunities for sustainable and industrial-scale applications [16,19].

Furthermore, the integration of omics-based approaches, including metabolomics, proteomics, transcriptomics, and bioinformatics, offers significant opportunities to advance *M. charantia* research [169]. Metabolomics can facilitate the identification and characterization of bioactive metabolites, while transcriptomics and proteomics can elucidate molecular targets, signaling pathways, and mechanisms underlying biological activities. These methods further support biomarker discovery, predict biological interactions, and accelerate the development of functional foods, nutraceuticals, and pharmaceutical products derived from *M. charantia* [170]. In addition, innovative formulation strategies such as nanoencapsulation, microencapsulation, and targeted delivery systems should be explored to enhance bioavailability, improve stability, and increase consumer acceptance.

Moreover, well-designed preclinical studies and multicenter clinical trials are also needed to establish therapeutic efficacy, optimal dosage, bioavailability, and safety in humans. In addition, advanced delivery systems such as nanoencapsulation, microencapsulation, and controlled release formulations may enhance stability, bioavailability, and sensory acceptance. Industrial applications may extend beyond functional foods and nutraceuticals to pharmaceuticals, biodegradable packaging, and natural preservatives. Utilizing underexplored by-products such as seeds, peels, and leaves could further support waste valorization, contributing to a circular bioeconomy and more sustainable food systems.

## 9. Conclusions

*M. charantia* is a nutritionally rich plant containing diverse bioactive compounds, including charantin, momordicosides, cucurbitacins, phenolics, flavonoids, saponins, and triterpenoids, that contribute to its antidiabetic, antioxidant, anti-inflammatory, hepatoprotective, and anticancer properties. Among the extraction approaches evaluated, ultrasound-assisted extraction appears particularly promising because it improves the recovery of bioactive phytochemicals while reducing extraction time, solvent consumption, and environmental impact, making it suitable for large-scale applications. From an industrial perspective, *M. charantia* shows strong potential as a functional ingredient across a broad range of product categories, including functional foods and beverages, fortified bakery products and snacks, as well as nutraceutical and dietary supplement formulations. The increasing consumer demand for plant-based bioactives, natural antioxidants, and clean-label ingredients further supports its potential integration into modern food and health-related products. However, several challenges remain for its widespread commercial application. Variability in phytochemical composition due to cultivar, geographic origin, and processing conditions can affect product standardization and consistent efficacy, while the characteristic bitterness of *M. charantia* may limit sensory acceptance in food formulations. In addition, regulatory approval pathways for nutraceutical and functional food ingredients differ across regions and may require further toxicological, clinical, and quality-control evidence to ensure safety and efficacy. Future research should therefore focus on standardizing extraction processes, improving bioavailability and sensory properties through advanced formulation strategies (e.g., encapsulation or delivery systems), and conducting well-designed clinical studies to confirm therapeutic benefits. Moreover, valorization of underutilized plant parts such as seeds, peels, and leaves may enhance sustainability and support the development of innovative products within a circular bioeconomy. Overall, *M. charantia* represents a promising natural resource for the development of functional foods, nutraceuticals, and pharmaceutical products aimed at preventing chronic diseases and promoting human health, provided that technological, regulatory, and market-related challenges are addressed through continued interdisciplinary research.

## Figures and Tables

**Table 2:** Advanced extraction techniques and impact on the yield of bioactive compounds.

Extraction Method	Solvent Used	Conditions	Extract Yield	Extract Properties	References
Ultrasound-assisted extraction	Ethanol (30–90%)	Ultrasonic power: 150–300 W, extraction time: 5–30 min	33.42%	TPC: 18.73 mg GAE/g, DPPH: 66.93%, charantin: 28.56 mg/g extract	[49]
Ultrasound-assisted extraction (probe type)	Ethanol or distilled water (60% *v*/*v*)	Extraction time: 15 min, frequency: 20 kHz, ultrasonic intensity: 270 W	37.72%	TPC: 18.73 mg GAE/g, charantin: 28.56 mg/g, TFC: 8.29 mg NE/g	[50]
Ultrasound-assisted extract	Ethanol (80% *v*/*v*)	Acoustic intensity: 85 W·cm^−2^, duty cycle: 0.83 s^−1^, extraction time: 13.2 min	35.80%,	TPC: 847.91 mg GAE/100 g, DPPH: IC_50_: 119.08 µg/mL, FRAP: 148.76 mg AAE/g, charantin: 5.83 mg/g	[51]
Ultrasound-assisted extraction	Aqueous extract (0.25 g/mL bitter gourd to water ratio)	Temperature: 68.4 °C, time: 12 min	NN	Polyphenols: 104.5 mg GAE/g, protein: 46.2 mg/1000 mL, DPPH: 69.9%	[52]
Hot reflux	Aqueous ethanol (50% Ethanol in 500 mL water)	Temperature: 150 °C, time: 6 h	10.23 mg/50 g	DPPH free radical scavenging: 2.29 g Trolox/100 g	[53]
Pressurized liquid extraction	Acetone, dichloromethane, ethanol, water	Temperature: 120 °C, pressure: 10 MPa, time: 40 min, solvent 40 mL	96.05%	Charantin: 0.126 ± 0.018 mg/g	[54]
Soxhlet extraction	Ethanolic extract	Temperature: 78.5 °C, Pressure: 10 MPa, time: 150 min, and solvent: 200 mL	97.51%	Charantin: 0.24 mg/g	[54]
Supercritical fluid extraction	SC-CO_2_ with ethanol	Time: 2.5 h	1.924%	Charantin: (0.7817 mg/g)	[55]
Ultrasound-assisted extraction	Methanol: Water (80:20, *v*/*v*, solid to solvent ratio of 1:26 *w*/*v*)	Temperature: 46 °C, time: 120 min	3.18 mg/g	Charantin: 3.12 mg/g	[57]
Ultrasonic-assisted extraction	Aqueous ethanolic solvent	Extraction time (20, 40, 60 min), temperature (30, 45, 60 °C), induced calorimetric power (38.50, 53.25, 68.00 W)	28%	TPC: 0.95 ± 0.11 g GAE/100 g, TFC: 0.32 ± 0.03 g QE/100 g, FRAP: 109.00 109.00 µmol Fe(II) eq./g, DPPH: 22.0 mg/mL	[58]
Soxhlet extraction	Methanolic extract	Temperature: 70 °C	15 mg/35 kg	NN	[59]

Abbreviations used: DPPH, 2,2-diphenyl-1-picrylhydrazyl; FRAP, ferric reducing antioxidant power; NE, naringin equivalents; NN, not known; QE, quercitrin equivalents; TFC, total flavonoid content; TPC, total phenolic content.

**Table 3:** Targeted mechanisms and potential benefits of *M. charantia* against specific cancer cell lines.

Cancer Type	Cell Line	Model	Dose/IC_50_	Targeted Mechanism	Potential Benefits	Reference
Bladder cancer	BC 5637 and T24	In vitro/In vivo	1.25, 2.50, 5.00, 10.00, 20.00, 40.00, and 60.00 µg/mL	↓ CENP-A, ↑ p21, p16, apoptosis, senescence	Suppressed proliferation, migration, tumor growth in vivo	[61]
Cervical cancer	HeLa, C33A	In vitro/In vivo	IC_50_: 59–68 µg/mL	↓ PCNA, Cyclin D1, p-Akt; ↑ Bax; ferroptosis (GPX4)	Reduced proliferation, migration, induced apoptosis, ferroptosis, inhibited tumor growth	[63]
Head and neck cancer	HNC	In vitro/In vivo	Not specified	↓ GLUT1, HK1, PFKP, LDHA; ↓ FASN, SREBP1; AMPK↑, mTOR↓	Reduced tumor metabolism, induced autophagy, apoptosis, decreased tumor size	[59]
Melanoma	B16	In vivo	Oral extract	↓ PAX3, ↓ AKT/mTOR, ↓ BCL-2	Reduced proliferation, lung metastasis	[64]
Liver cancer	SK-Hep-1	In vitro	Not specified	↑ ROS, ↓ PARP-1, Caspase-3/9, mitochondrial apoptosis	Induced apoptosis, G1 arrest, mitochondrial dysfunction	[62]
Multiple cancers	MCF-7, Hep-G2, WiDr	In vitro	IC_50_: 27–40 µM	Moderate cytotoxicity via triterpenoids	Moderate anti-inflammatory, weak antiproliferative activity	[106]
Liver cancer	HCCLM3	In vitro	Dose-dependent	↑ ROS, ↓ PARP-1, Caspase-3; cell-cycle arrest	Reduced proliferation, induced apoptosis and G0/G1 arrest	[107]
Oral cancer	KB	In vitro	Not specified	↑ Antioxidant enzymes, ↓ mutant p53 signaling	Strong antiproliferative, antioxidant activity	[108]
Breast, liver, colon	MCF-7, Hep-G2	In vitro	IC_50_: ~14–20 µM	Cytotoxic triterpenoids (kuguacin derivatives)	Significant cytotoxicity and anti-inflammatory effects	[109]
Oral cancer	OSCC	In vitro/In vivo	Not specified	↓ NLRP3, ↑ ROS-mediated apoptosis; reduces drug resistance	Enhanced chemotherapy (5-FU), reduced drug resistance	[110]
Pancreatic cancer	PANC-1	In vitro/In vivo	Saponins rich fractions (5%, 10%, 15%)	Decreased the total cell number, induce strong cell death in PANC-1 cells	inhibit PANC cell proliferation and induce cell death; suppress PANC tumor growth, proliferation, induce apoptosis, restrict capillary tube formation by human umbilical vein endothelial cells, decrease angiogenesis in PANC tumor xenografts	[101]
Lung cancer	A549	In vivo/In vitro	IC_50_: 17.3 ± 0.01 μg/mL	Increased the caspase-3/7 activity by 1.6-fold, ROS activity by 5-fold	Decrease in cell viability, ROS-mediated apoptosis, inhibition of tumor cell proliferation, acceleration of the rate of tumor cell death, and/or induction of tumor cell differentiation	[96]
Multiple cancer cell lines	MCF-7, HEp-2, HepG2, WiDr	Mice model	Lyophilized powder (1%, 2%, 10%)	Anti-inflammatory (NO inhibition), weak cytotoxicity	Moderate anti-inflammatory, weak antiproliferative activity	[111]
Oral carcinoma	KB cells	In vivo	4-nitroquinoline 1-oxide (50 μg/mL), BME (30% *v*/*v*, 600 mg/mouse)	Antioxidant activity, tumor suppressor gene modulation (p53, TGF-β)	Strong antiproliferative, antioxidant activity	[112]

Abbreviations used: ACSL4, acyl-CoA synthetase long chain family member 4; AKT/mTOR, protein kinase B/mammalian target of rapamycin; AMPK, AMP-activated protein kinase; Bax, Bcl-2-associated X protein; BC cells, bladder cancer cells; BCL-2/Bcl-2, B-cell lymphoma 2; CENP-A, centromere protein A; FASN, fatty acid synthase; GLUT1, glucose transporter type 1; GPX4, glutathione peroxidase 4; HCCLM3, human hepatocellular carcinoma cell line; HK1, hexokinase 1; HNC cells, head and neck cancer cells; IC_50_, half maximal inhibitory concentration; LDHA, lactate dehydrogenase A; mTOR, mammalian target of rapamycin; NLRP3, NOD-like receptor family pyrin domain containing 3; OSCC, oral squamous cell carcinoma; PARP-1, poly ADP-ribose polymerase-1; PAX3, paired box 3; p-Akt, phosphorylated protein kinase B; PCNA, proliferating cell nuclear antigen; PFKP, phosphofructokinase; ROS, reactive oxygen species; SREBP1, sterol regulatory element binding protein 1. ↑, expression or concentration upregulated; ↓ expression or concentration downregulated.

## Data Availability

No new data were created or analyzed in this study.

## Abbreviations

The following abbreviations are used in this manuscript:

ABTS	2,2′-azino-bis(3-ethylbenzothiazoline-6-sulfonic acid)
ACSL4	Acyl-CoA synthetase long chain family member 4
ACC	Acetyl-CoA carboxylase
AKT/mTOR	Protein kinase B/mammalian target of rapamycin
AMPK	AMP-activated protein kinase
ARE	Antioxidant response element
BAX	BCL-2-associated X protein
BC cells	Bladder cancer cells
BCL-2	B-cell lymphoma 2
CAT	Catalase
CDK1	Cyclin-dependent kinase 1
CDK2	Cyclin-dependent kinase 2
CDK4/6	Cyclin-dependent kinases 4 and 6
CENP-A	Centromere protein A
ChREBP	Carbohydrate-responsive element-binding protein
CiC	Citrate carrier
CPT-1	Carnitine palmitoyltransferase-1
DPPH	2,2-diphenyl-1-picrylhydrazyl
E2F	E2F transcription factor
GAP	GTPase-activating protein
GEF	Guanine nucleotide exchange factor
GLP-1	Glucagon-like peptide-1
GLUT1	Glucose transporter type 1
GLUT4	Glucose transporter type 4
GLUT5	Glucose transporter type 5
GPX4	Glutathione peroxidase 4
GSH	Reduced glutathione
GDP	Guanosine diphosphate
GTP	Guanosine triphosphate
HCCLM3	Human hepatocellular carcinoma cell line
HK1	Hexokinase 1
HNC cells	Head and neck cancer cells
IC50	Half maximal inhibitory concentration
IFN-γ	Interferon gamma
IKKα/β/γ	Inhibitor of kappa B kinase subunits alpha, beta, gamma
IκB	Inhibitor of kappa B
IL-1β	Interleukin-1 beta
IL-6	Interleukin-6
IL-8	Interleukin-8
IL-10	Interleukin-10
IRS-1	Insulin receptor substrate 1
KRAS	Kirsten rat sarcoma viral oncogene homolog
LDHA	Lactate dehydrogenase A
LRR	Leucine-rich repeat
MAPK	Mitogen-activated protein kinase
mTOR	Mammalian target of rapamycin
mTORC1	Mechanistic target of rapamycin complex 1
NACHT	NAIP, CIITA, HET-E, and TP1 domain
NF-κB	Nuclear factor kappa-light-chain-enhancer of activated B cells
NLRP3	NOD-like receptor family pyrin domain containing 3
Nrf2	Nuclear factor erythroid 2–related factor 2
OSCC	Oral squamous cell carcinoma
PAX3	Paired box 3
PARP-1	Poly ADP-ribose polymerase-1
PCNA	Proliferating cell nuclear antigen
PFKP	Phosphofructokinase
PI3K	Phosphoinositide 3-kinase
PPAR-α	Peroxisome proliferator-activated receptor alpha
PPAR-γ	Peroxisome proliferator-activated receptor gamma
PYD	Pyrin domain
RB	Retinoblastoma protein
RNS	Reactive nitrogen species
ROS	Reactive oxygen species
SIRT1	Sirtuin 1
SOD	Superoxide dismutase
SREBP1	Sterol regulatory element binding protein 1
STAT1	Signal transducer and activator of transcription 1
STAT3	Signal transducer and activator of transcription 3
sMaf	Small musculoaponeurotic fibrosarcoma protein
TAB1/2/3	TAK1-binding proteins 1, 2, and 3
TAK1	Transforming growth factor-β activated kinase 1
TFC	Total flavonoid content
TLR	Toll-like receptor
TNF-α	Tumor necrosis factor alpha
TPC	Total phenolic content
TTC	Total tannin content
Treg	Regulatory T cells
Th1	T helper 1 cells
Th17	T helper 17 cells
UAE	Ultrasound-assisted extraction
